# Cutaneous Adverse Reactions Associated with Tattoos and Permanent Makeup Pigments

**DOI:** 10.3390/jcm13020503

**Published:** 2024-01-16

**Authors:** Beatrice Bălăceanu-Gurău, Eliza Apostol, Mădălina Caraivan, Ana Ion, Raluca Tatar, Mara Mădălina Mihai, Liliana Gabriela Popa, Cristian-Dorin Gurău, Olguța Anca Orzan

**Affiliations:** 1 Department of Oncologic Dermatology, “Elias” Emergency University Hospital, “Carol Davila” University of Medicine and Pharmacy, 020021 Bucharest, Romania; balaceanubeatrice@yahoo.com (B.B.-G.); alina-eliza.apostol@rez.umfcd.ro (E.A.); anaion00@yahoo.com (A.I.); mara.mihai@umfcd.ro (M.M.M.); liliana.popa@umfcd.ro (L.G.P.); olguta.orzan@umfcd.ro (O.A.O.); 2 Clinic of Dermatology, “Elias” Emergency University Hospital, 011461 Bucharest, Romania; 3 Private Practice, 1430 Luxembourg, Luxembourg; 4 Department of Plastic Reconstructive Surgery and Burns, “Grigore Alexandrescu” Clinical Emergency Hospital for Children, “Carol Davila” University of Medicine and Pharmacy, 020021 Bucharest, Romania; 5 Department of Plastic Reconstructive Surgery and Burns, “Grigore Alexandrescu” Clinical Emergency Hospital for Children, 010621 Bucharest, Romania; 6 Orthopedics and Traumatology Clinic, Clinical Emergency Hospital, 014451 Bucharest, Romania; gurau_dorin@yahoo.com

**Keywords:** tattoos, permanent makeup, cutaneous adverse reactions, tattoo-related complications

## Abstract

Tattooing is the procedure of implanting permanent pigment granules and additives into the dermal layer of the skin, serving various purposes such as decoration, medical identification, or accidental markings. There has been a significant rise in the popularity of decorative tattooing as a form of body art among both teenagers and young adults. Thus, the incidence of tattoos is increasing, with expanding applications such as permanent makeup, scar camouflage, nipple–areola, lips, and eyebrows tattooing, and utilization in oncological radiotherapy such as colon marking. However, there have been reported a broad range of adverse reactions linked to tattooing, encompassing allergic reactions, superficial and deep cutaneous infections, autoimmune disorders induced by the Koebner phenomenon, cutaneous tumors, and others. These reactions exhibit different onset times for symptoms, ranging from immediate manifestations after tattoo application to symptoms emerging several years later. Given the limited information on a tattoo’s side effects, this review aims to elucidate the clinical spectrum of cutaneous complications of tattoos in different patients. The analysis will investigate both allergic and nonallergic clinical presentations of tattoo-related side effects, microscopic findings from skin biopsies, and therapeutic outcomes. This exploration is essential to improve our understanding of tattoo-related cutaneous complications and associated differential diagnoses and highlight the significance of patient awareness regarding potential risks before getting a tattoo.

## 1. Introduction

The term “tattooing” is rooted in the Tahitian word “tattau”, meaning “to mark” [1]. It represents the procedure of implanting permanent pigment granules and additives into the dermal layer of the skin, serving various purposes such as cosmetic applications (decorative tattoos and permanent makeup) or therapeutic uses (medical tattoos) [1]. Medical therapeutic tattooing plays an important role in techniques such as camouflage for vitiligo, breast areola reconstruction after radical surgery, concealing permanent hair loss following craniofacial surgery, and addressing scars after plastic and reconstructive surgery [1]. Accidental occurrences, like traumatic tattoos resulting from abraded skin injuries, can also be encountered [1]. 

There has been a significant contemporary upswing in tattooing, particularly among teenagers and young adults, as a form of cosmetic and decorative body art [1]. Currently, there is a lack of stringent requirements, regulations, and legislative measures ensuring the safety of tattooing [1]. Consequently, the reported incidence of adverse reactions after tattooing has been increasing, although these are often observed by physicians but remain relatively unknown to the general public and tattoo artists [1]. The shift in tattoo-ink composition from inorganic pigments (heavy metals) to organic pigments (azo pigments) in recent decades and the subsequent use of postcare products adds another layer of complexity to understanding potential complications [2,3].

Common skin reactions documented in the medical literature encompass a transient acute inflammatory response resulting from skin trauma induced by needles, involving pain, development of blisters, crusts, and pinpoint hemorrhaging [2,3]. Moreover, there have been reported a wide range of emerging cutaneous manifestations. Skin conditions and issues following the process of tattooing can be classified into inflammatory disorders (allergic reactions, chronic inflammatory black tattoo reactions, autoimmune skin afflictions, foreign-body reactions, and pseudo lymphoma), infections (bacterial, mycobacterial, viral, fungal, and parasitic), neoplasms (benign and malignant tumors), miscellaneous complications (neuro-sensory issues, complications linked to magnetic resonance imaging, and photoinduced reactions) and cosmetic issues (misapplication, pigment fanning or migration, and scars) [1,2,3].

Delayed complications may include, in addition to scarring and cutaneous textural changes, pigmentary alterations associated with tattoo removal using Q-switched lasers, such as hypopigmentation and hyperpigmentation, and the occurrence of paradoxical darkening of the tattooed area or residual pigmentation [1].

Our review seeks to offer a thorough description of the various types, clinical manifestations, and, when applicable, microscopic findings of dermatological complications linked to tattooing, along with their occurrence and underlying conditions. This investigation is essential for advancing our understanding of cutaneous complications associated with tattoos, including their differential diagnoses and therapeutic approaches.

Additionally, we underscore the importance of raising patient awareness about potential risks before deciding to get a tattoo. Individuals with various skin diseases should be warned of the potential risk of localization of specific cutaneous afflictions in a tattoo. Moreover, adverse reactions to tattoos may also be the initial presentation of a specific skin affliction.

## 2. Tattoo Trends and Practices

Throughout tattooing history, various methods and techniques have been devised to achieve permanent body modifications, ranging from traditional, deeply rooted methods to more contemporary approaches.

Piercing, the most prevalent method among modern tattoo artists involves using an object like a needle to push ink into the skin [4]. Puncturing necessitates substantial force to break through the skin, typically achieved with a tattooing device held at a 90-degree angle whereas the cutting method involves tools dipped in ink to make incisions into the skin [4].

Hand poking stands as the oldest tattooing technique [4]. In this approach, a single needle is employed to puncture the skin, creating dots of ink, and is generally associated with minimal pain [4]. Yantra and Tebori are also historic techniques that work by puncturing the skin with a bamboo stick dipped in ink [4].

The stick-and-poke technique is frequently carried out at home, with individuals applying the method on themselves by repetitively piercing the needle into the skin and commonly utilizing various available inks [4].

The single-needle technique employs the method of piercing and utilizes a single needle, driven by a tattoo machine [4]. Tattoos created through the single-needle technique are typically small, intricate, and monochromatic [4].

Currently, tattoo artists have access to various machines and needles, and employing the right products is crucial in this process; high-quality products do make a significant difference [4]. A pricier machine ensures precise and uniform ink transfer into the customer’s skin compared to one that operates irregularly and poses a risk of skin damage [4].

The same aforementioned principle extends to needles and colors. More professional needles obtained from reputable manufacturers offer superior performance and safety [4]. While some other needles might work, the inconsistency in quality makes it challenging for tattoo artists to consistently produce high-quality work [4].

The composition of elements in tattoo inks exhibits significant variation, even among pigments of similar colors [5,6]. This underscores the intricate and diverse chemical composition of tattoo inks, emphasizing the need for an accurate diagnosis and treatment [6]. The green dye utilized in tattoos is commonly composed of chromium oxide, lead chromate, phthalocyanine dyes, ferrocyanides, and ferricyanides [6]. Cobalt is typically responsible for the blue color, while cadmium sulfide contributes to the yellow pigment [6]. Cinnabar, a mercury sulfide, ferric hydrate (sienna), sandalwood, brazilwood, and iron oxide contribute to the red color, and manganese and aluminum to the purple color [6]. Mercury, chromium, cadmium, and cobalt have been reported to induce various types of reactions in sensitized individuals [7,8].

In recent decades, mineral pigments have seen widespread substitution by vibrant organic pigments, notably falling into chemical classes such as azo pigments, quinacridones, and phthalocyanines [8]. Case reports suggest that the former two classes may act as sensitizers and contribute to allergies, particularly in tattoos with red hues [8]. However, many studies struggle to establish a direct link between allergic reactions and organic pigments [8]. This challenge arises because reports often rely on ingredient lists on ink bottles without verifying pigments through chemical analysis in the ink or the patient’s skin [8]. Notably, approximately one-third of ink labels provide inaccurate information about the pigments used [8].

Pigments P.R. 22, P.R. 210, and P.R. 170 have been identified as the predominant contributors to chronic allergic reactions in red tattoos [8]. The epitope responsible for the reaction might be a degradation product of the pigment [8]. The presence of metal contamination, originating from various sources, and its role in red tattoo allergies was not conclusively determined [8].

According to the existing literature, tattoo inks commonly contain a diverse array of metals [8]. Concerning tattoo safety and the risk of allergic sensitization, nickel and chromium are particularly relevant [8]. A prior investigation involving allergy-patch testing in patients with chronic tattoo reactions, encompassing allergies related to red tattoos and instances of cross reaction, revealed a positive reaction to nickel sulfate in 21% of the cases [8].

Lately, there has been a surge in studies exploring the application of medical tattooing to achieve three-dimensional effects [9]. While the predominant use of medical tattooing in this context is for the restoration of the nipple–areola complex, various other applications of three-dimensional tattooing are coming to the forefront [9]. Concurrently, there is a growing reliance on nurses and medical tattoo assistants to carry out these procedures [9].

## 3. Cutaneous Adverse Reactions

A tattoo invariably triggers an acute aseptic inflammatory response of varying intensity [5,6,7]. This reaction is characterized by erythema, induration, and an edematous appearance, accompanied by the dilation of hair follicles [7]. This immediate response occurs during the tattooing session, right after the needle punctures the skin [7]. The freshly inked tattooed area becomes surrounded by tender, erythematous, and red borders [7]. Depending on the individual’s skin, petechial purpura might be visible as well [7]. Over the next 2–3 weeks, the tattoo gradually heals, with superficial crusts forming within a week [7]. The retained ink in the epidermis is shed as the outer layer peels away [7]. Acute transient lymphadenopathy may occur in the draining area of the tattoo during the healing process [7].

In medical practice, cutaneous complications are classified based on a comprehensive evaluation, taking into consideration the patient’s history, clinical manifestation of the tattoo reaction, a thorough physical examination, and histopathology of the reaction.

### 3.1. Inflammatory Tattoo-Related Side Effects

#### 3.1.1. Allergic Reactions

##### Definition and Pathophysiology

Hypersensitivity reaction to tattoos or tattoo allergy has been described as a chronic and persistent reaction affecting one or more tattoos, limited to a single color, and manifesting over variable periods, from onset to several years after tattoo completion [5]. Allergic reactions to tattoos are the most prevalent complication, as other studies previously stated [5,6] (Table 1).

Allergic reactions to tattoos are primarily believed to be T-cell mediated, falling under the category of delayed type-IV hypersensitivity [10]. The progression of a type-IV hypersensitivity reaction typically consists of a sensitization phase and an elicitation phase [10].

Limited studies propose the involvement of antibodies in chemical-induced hypersensitivity adverse reactions [10,14]. In the context of tattoos, few clinical cases were reported to likely encompass reactions mediated by antibodies [10,15,16,17]. A case of anaphylaxis in a patient who had previous sensitization to colored ink was also mentioned [18,19]. Although considered a rare reaction, it is advisable to acknowledge it [18,19].

##### Clinical Manifestations

**Figure 1:**
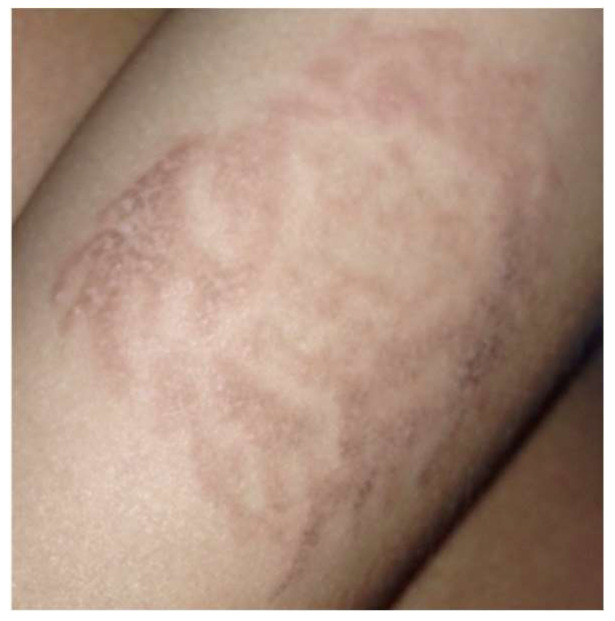
Allergic contact dermatitis developed on the left forearm after the application of a sticker.

Typically, allergic contact dermatitis (ACD) presents approximately 1–3 days after contact with a topical chemical and subsides upon removal of the triggering allergen [10]. The combination of henna tattoos with p-phenylenediamine (PPD) to achieve a black coloration can also lead to the development of ACD (Figure 1). This reaction may be accompanied by systemic manifestations, such as generalized lymphadenopathy and fever [20].

Clinical presentations of allergic cutaneous reactions include papulonodular, plaque-like, lichenoid, hyperkeratotic, or ulceronecrotic patterns [2,10]. Allergic reactions often manifest locally within the entire tattooed area with the triggering color, but generalized rashes or eczemas have been reported, especially in previously sensitized individuals [10]. These reactions usually resolve without treatment after a few weeks or months, suggesting the involvement of soluble tattoo-ink components [10].

Generally, symptoms of an allergic reaction to tattoo pigments often lack specificity and can manifest as tenderness, localized or diffuse swelling, and asymptomatic or itchy papules or nodules, accompanied by crusts and excoriation due to isolated pruritus [7]. Even though itch is a commonly encountered symptom, pain has rarely been noticed [7].

Red tattoo pigments are the most commonly involved in allergic reactions [2]. In red tattoos, allergic responses may include itching, swelling, eczematous, granulomatous, and sarcoidal reactions [21]. Contact urticaria-like reactions or photoallergic reactions may also be observed, particularly in older tattoos [10].

A case of a generalized eczematous eruption following the laceration of a tattoo in a patient with sensitivity to mercury has also been documented [22].

##### Potential Trigger Factors

Allergic reactions without generalized rashes can be initiated by laser treatment and potentially by sun exposure, leading to a sensitizer’s release, a phenomenon termed photoallergy, distinct from phototoxicity [10]. As a result, sunlight can be a trigger, with light capable of inducing the chemical cleavage of tattoo pigments, potentially contributing to allergen formation [2].

Generalized reactions may occur during attempts to remove pigment with laser treatment as well or due to photoallergic reactions in tattoos with yellow ink [10].

In rare cases, allergic reactions in tattoos may be triggered by implant materials, leading to implant removal [10]. 

Although the precise allergen usually remains unidentified, one study by Serup et al. suggested the likely involvement of naphthol AS azo pigments [8].

**Figure 2:**
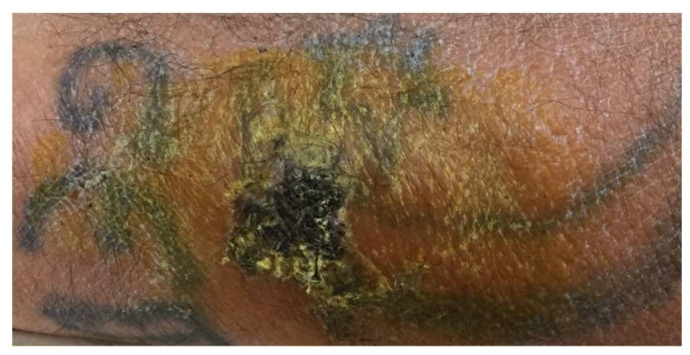
Clinical image of a contact dermatitis case developed on the right forearm.

Nevertheless, in the context of tattooing, allergic reactions extend beyond tattoo pigments [1]. Latex gloves worn by tattoo artists can induce severe type-I allergic reactions [1]. Furthermore, some reports mentioned the existence of type-IV allergic reactions associated with tattoo aftercare products (fragrance, wool alcohols, panthenol, and colophony) [1]. Consequently, there is a potential for acute contact dermatitis in sensitized individuals, particularly in response to topical agents such as disinfectants, which can delay the healing process [7] (Figure 2).

##### Chronic Inflammatory Black Tattoo Reactions (CIBTR)

In tattoos, the potential allergen might be permanently present, potentially leading to chronic inflammation. Delayed-type hypersensitivity reactions associated with tattoos can occur shortly after application or several years later, depending on individual sensitization and the need for metabolism, degradation, or additional immune stimulation for allergen initiation [7].

Chronic reactions to tattoo pigments lead to the development of fibrosis and granulomatous changes, contributing to the persistence of skin alterations [7]. This chronicity is associated with the formation of nodules and fibrotic tissue in the dermis, indicating a prolonged inflammatory response to the tattoo [7].

CIBTR, also known as ‘papulo-nodular’ reactions, clinically manifest as persistent papules or nodules, localized exclusively to the black-inked skin, regardless of whether they originate from sarcoidosis or not, and devoid of any clinical or histological indications of concomitant infection [2]. Typically, clinical symptoms involve mild pruritus or pain [2]. Such reactions are acknowledged as potential indicators of sarcoidosis; although the majority of CIBTRs are categorized as nonsarcoidosis, as they typically lack concurrent manifestations associated with sarcoidosis (hilar lymphadenopathy, erythema nodosum, lupus pernio, uveitis, and Lofgren’s syndrome) [2,3,23,24,25]. Solely relying on the cutaneous clinical and histological characteristics may not always permit differentiation [2]. Some authors argue that these reactions are nonallergic, attributing this to the inert nature of the primary pigment in black tattoos [26]. Nonetheless, tattoo inks may contain other pigments, including heavy metals, leading to the ongoing debate about the role of heavy metals [26].

Moreover, the manganese present in purple tattoos has been identified as a causative factor for granulomatous reactions in specific patients [7]. Additionally, tattoo-associated dermatoses, such as lichenoid reactions, pseudo lymphomas, and morphea-like lesions, may manifest as part of the immune response to the pigments, further complicating the clinical presentation [7,27,28].

##### Diagnostic Approaches

A potential diagnostic strategy for tattoo-related allergies involves obtaining a detailed clinical case history to gather information on the suspected allergen’s characteristics, such as its association with pigments and exposure to light or soluble ink ingredients [10].

Patch testing is considered the gold standard in the case of ACD, but it frequently yields negative results, possibly due to the challenge of obtaining suitable patch-test solutions given the limited dispersing capacities of most pigments [1,10]. Patch testing, previously performed mainly on patients with reactions to red pigment in tattoos, showed negative results, while others presented positive reactions to various acrylates and a mixture of thiuram [6]. To enhance positive patch-test reactions, recommendations include tape stripping, delayed readings, and photopatch testing, particularly for red pigments [1].

In cases where a patient has encountered a specific reaction to a tattoo color, it is advisable to avoid using the same color in future tattoos, even if the ink brand may be different [7]. However, it is important to note that a positive patch test might indicate coincidental sensitization to the tested substance, and another allergen could be responsible for the skin reaction [10]. Additionally, individuals should be cautioned that predicting reactions to other colors is uncertain, as there might be a common substance present in both inks [7].

The limitation of patch testing lies in its ability to establish a correlation between sensitization and a substance the patient came into contact with, such as nickel sensitization correlating with nickel in tattoo ink [10]. Patch tests come with the uncertainty that other substances present in the tattoo ink might have been the primary cause of the allergic reaction [10].

A definitive diagnosis requires a pathological examination of a skin sample, and various histopathological patterns aid in a more precise classification, providing clues to potential conditions such as sarcoidosis or lichen planus (LP). González-Villanueva et al. proposed a diagnostic algorithm to guide dermatologists in the assessment of diverse reactions to tattoos and the prescription of a more appropriate medical intervention [29]. Treatment choice may be influenced by histopathological findings.

Chemical analysis, combined with examining the presence of an allergen in the biopsy or ink and conducting a positive patch test, may offer clues about the allergen’s identity [10]. Moreover, in vitro methods can reveal pathogenic T-cell populations in the patient compared to nonallergic controls [10]. Detecting increased numbers of allergen-reactive T-cell clones in the inflamed tattoo would strongly suggest a type-IV hypersensitivity reaction to a substance present in the biopsy or ink [10]. Utilizing high-throughput sequencing technologies can provide conclusive evidence that the skin reaction is indeed caused by the suspected substance [10].

##### Histopathologic Features

**Figure 3:**
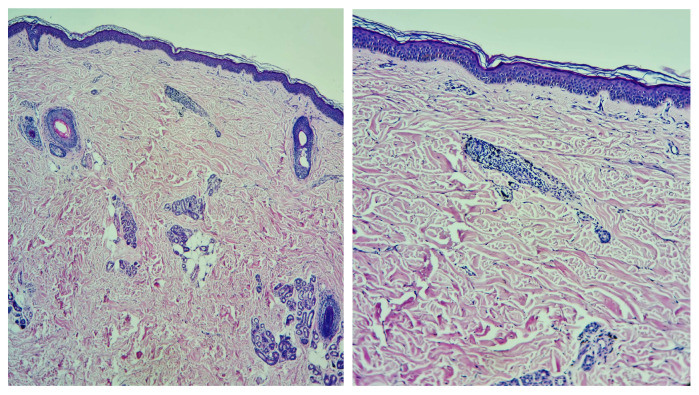
Hematoxylin–eosin staining, an inflammatory infiltrate with lymphocytes and collections of macrophages loaded with black pigment (tattoo pigment), is evident and arranged superficially perivascular in the papillary dermis.

Histologically, allergic reactions to tattoos have consisted of epidermal reactions (acanthosis, hyperkeratosis, or parakeratosis) associated with a persistent inflammatory infiltrate in the dermis, mainly composed of lymphocytes [1,5,30] (Figure 3). Additional cellular infiltrates, including macrophages, histiocytes, plasma cells, eosinophils, or neutrophils, have been variably present [5,30]. Granulomas have been occasionally observed, either in association with epidermal reactions or independently [5,30].

The primary diagnosis can be classified depending on the predominant reaction observed following biopsy [1,5,31]:

- lichenoid reactions;
- granulomatous reactions (foreign-body granuloma; sarcoidal granuloma);
- cutaneous lymphoid hyperplasia (pseudo lymphoma);
- eczematous reactions.

Lichenoid reactions are primarily associated with red ink [3]. These reactions can be attributed either to an allergy or to LP, characterized by planar polygonal papules and plaques [3]. A localized flare up of LP is typically linked to the Köbner phenomenon and thus not necessarily limited to a specific color [3]. However, distinguishing clinically and histologically between lichen planus and allergic reactions with a lichenoid infiltrate remains challenging. Allergic reactions typically affect a single color, though the reason for this specificity remains unclear given the diversity of ink compositions [3]. Cases where a localized lichenoid reaction on tattooed skin progressed to a generalized lichenoid reaction have also been mentioned [3]. There is still some uncertainty regarding whether the lichenoid reaction is triggered by the Köbner phenomenon in an (undiagnosed) LP patient or if it is a result of the tattoo ink in an allergic patient, as the exact etiology remains unknown. Topical tacrolimus is useful in case of lichenoid reactions [1].

Granulomatous inflammation is a common occurrence [23]. A foreign-body reaction to pigment can give rise to elevated red bumps at the tattoo site, comprising epithelioid cells, lymphocytes, and occasional giant cells [23]. Granulomatous reactions have been linked to tattoos featuring various pigments, including red, green, blue, purple, and ultraviolet (UV) visible inks [23]. The differential diagnosis for noninfectious granulomatous reactions includes allergic reactions, foreign-body reactions, and sarcoidosis [3].

Sarcoidosis manifested on tattoos is most appropriately classified as ‘scar sarcoidosis’, a well-recognized complication [3,11,12,32,33]. Sarcoidosis has been documented in tattoos of various colors, with black ink being the most commonly reported [3,7,34]. It may appear months or even years after placement [3,7]. Chronic exposure of the immune system to ink may stimulate granuloma development and potentially lead to granulomatous inflammation in individuals genetically predisposed to sarcoidosis [2,3,7].

Instances where cutaneous sarcoidosis is confined to a singular pigment color often lead to the debate about whether it represents a real sarcoidal hypersensitivity reaction to the exogenous pigment or serves as the first (and solitary) manifestation of an underlying systemic disease [1,3,11,12,32,33]. Some reported cases describe granulomatous tattoo reactions associated with uveitis, where inflammation is confined to the tattooed skin and eyes, and no other systemic features, particularly sarcoidosis, are identified [3]. As a result, Kluger et al. introduced the term “Tattoo Granulomas with Uveitis” (TAGU) to describe cases where alternative diagnoses are excluded, and there is insufficient evidence to diagnose sarcoidosis [10,34].

Symptoms manifesting up to several years after tattoo placement may suggest a potential contribution from additional triggering factors, such as infection or certain systemic medications (targeted therapies, BRAF and MEK inhibitors, allopurinol, and antiretroviral therapy) [35]. Since granulomatous skin reactions may serve as the initial presentation of systemic disease, patients should undergo a comprehensive screening for sarcoidosis, encompassing chest imaging and laboratory tests (analysis of angiotensin-converting enzyme (ACE) and soluble interleukin-2 receptor (sIL2r) levels) and examination for ocular involvement [2,3,7,34].

Foreign-body reactions frequently occur in the borders or corners of a tattoo, where pigment density is higher than in other areas of the tattoo [3,7]. Touch ups are believed to increase the risk of pigment overload and, consequently, lead to a foreign-body reaction [3].

The diagnosis of pseudoepitheliomatous hyperplasia was attributed to cases where the intensity of epidermal hyperplasia was evident [5,31]. If the reaction is limited to one color, an allergic response is more probable [3]. When multiple tattoo colors are involved, distinguishing between an allergic reaction and a genuine pseudo lymphoma becomes challenging [3]. The precise pathogenesis of pseudo lymphoma remains unknown, although it is hypothesized that pigment induces chronic inflammation, leading to the polyclonal proliferation of lymphoid B and T cells [3]. Histologically, these reactions may resemble cutaneous T- or B-cell lymphoma, but they exhibit benign clinical behavior [3]. Malignant transformation is rare; however, one case reported the progression into a histologically malignant and immunologically monoclonal B large-cell lymphoma within a tattoo [3].

##### Differential Diagnosis

Differential diagnosis of type-IV hypersensitivity reactions to tattoos should consider other late reactions such as granulomatous foreign-body reactions, systemic diseases (sarcoidosis and connective tissue afflictions), microbial infections, and pseudolymphomatoid reactions [10,23].

##### Treatment Considerations

Different therapeutic strategies have been proposed throughout the scientific literature and are useful for dermatologists or any other clinician facing tattoo-related allergic reactions. Hypersensitivity reactions may resolve spontaneously, persist, or fluctuate over time [20]. Treatment approaches can vary from conservative measures to more invasive procedures, depending on the severity and location of the lesions (Table 1). Conservative treatment may involve the use of topical, oral, and/or intralesional steroids, oral antihistamines, and sunlight-exposure avoidance of the tattooed area [1]. The management of allergic reactions to tattoos has mainly involved the initial application of local corticosteroid (CS) ointments, especially those with high potency (clobetasol propionate), with or without occlusives [1,5]. Half of the patients reported a complete or partial response to this treatment, while medium-potency CS consistently proved ineffective [5]. Intralesional CS injections demonstrated efficacy in half of the cases, even in instances of significant reactions [5].

Invasive methods, while effective, carry the risk of causing permanent skin damage through scar formation [1]. These methods include cryotherapy, electrosurgery, surgical excision of the tattoo, dermabrasion, chemical destruction using acids, or ablation with a non-Q-switched carbon dioxide laser [1]. Q-switched lasers, CO_2_ ablative lasers, and Erbium lasers have also been proposed as therapeutic tools [36]. It is important to note that Q-switched laser therapy is not recommended when tattoos exhibit signs of an allergic reaction, as the therapy itself can trigger or exacerbate hypersensitivity reactions [36]. Nevertheless, the neodymium: yttrium–aluminum–garnet and Q-switched ruby lasers specifically aim at intracellular tattoo pigment [21]. They induce swift thermal expansion, leading to the fragmentation of cells containing the pigment and resulting in the dispersion of the pigment into the extracellular space [21]. Subsequently, the immune system identifies this extracellular pigment as foreign, leading to allergic reactions after laser treatment in some cases [21].

Notably, punch biopsies can selectively remove red, inflamed, and pruritic areas in certain cases.

#### 3.1.2. Autoimmune Dermatoses and Autoinflammatory Afflictions

**Figure 4:**
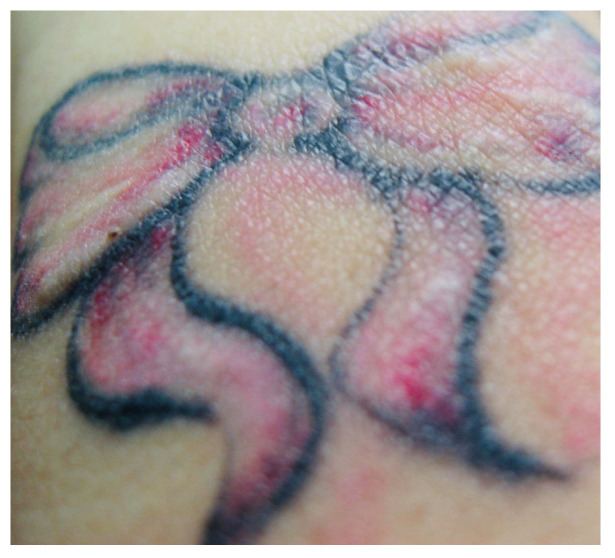
Granuloma annulare developed on a young adult’s tattooed wrist.

Individuals with chronic autoimmune skin conditions, including psoriasis, vitiligo, atopic dermatitis, LP, lichen sclerosus, pyoderma gangrenosum, lupus erythematosus, Darier’s disease, sarcoidosis, and autoinflammatory afflictions (granuloma annulare, morphea) face a risk of the skin disease localizing within a tattoo [3,7,11,12,13] (Figure 4 and Table 1). These skin disorders share a common trait wherein local skin trauma can trigger the onset of the disease, known as the Köbner phenomenon [3,7,11,12,13]. The trauma induced by the tattooing process triggers an inflammatory response, leading to localized exacerbations of these dermatoses [37,38,39].

**Figure 5:**
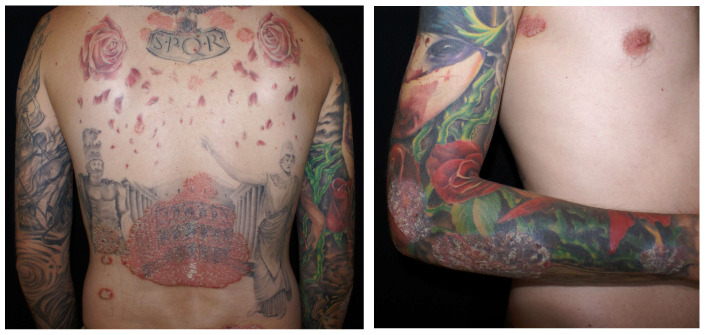
Psoriatic papules and plaques in a young man’s tattooed back, elbow, and forearm.

Heinrich Köbner initially described the Köbner phenomenon, observing the formation of psoriasiform lesions, notably in a recently tattooed area, in a patient with psoriasis [3,7,40,41]. The likelihood of psoriasis localizing in tattoos is influenced by the individual’s genetic predisposition and the activity level of the disease during the tattooing process [3,7,40,41] (Figure 5). While generalized flare-ups have been reported post-tattooing, establishing a definitive link remains uncertain [3,7,40,41]. Individuals with psoriasis should be cautioned about the potential for the disease to localize within a tattooed area.

Nevertheless, the scientific literature indicates a considerable variability in the time interval between tattooing and the onset of symptoms. For instance, Horner et al. reported a case of new-onset psoriasis guttate in a tattoo that manifested seven months after its placement, suggesting that an older skin trauma could also trigger an isomorphic inflammatory response [3,40].

Numerous cases of chronic lupus and, to a lesser extent, subcutaneous lupus linked to tattoos have been reported [42,43]. The lesions typically exhibit characteristic lupus features and may manifest as solitary entities or in association with multiple cutaneous lesions [42,43]. Remarkably, these lesions tend to manifest predominantly in the red areas of the tattoo [42,43]. The suspected pathophysiological mechanism in some cases involves the potential synergistic effect of UV light, particularly when combined with specific tattoo ink [42,43].

Clinical cases of LP have been correlated with localization to the tattoo sites [43]. When encountering a lichenoid reaction to a tattoo, it is important to rule out oral or cutaneous LP [43,44].

Finally, it is not uncommon for these cutaneous tattoo reactions to serve as the initial presentation of the underlying disease [37,38,39]. Individuals with autoimmune skin conditions should be aware of these potential adverse reactions before undergoing tattoo procedures [37,38,39].

A comprehensive clinical history and examination are imperative for accurate diagnosis. To confirm the diagnosis, a skin biopsy is essential, particularly when encountering papulonodular growth within the tattoo pigment [1]. If active disease is present, appropriate therapy should be administered in cases of koebnerization [1]. Standard treatment should be offered according to each affliction separately (Table 1).

### 3.2. Infectious Tattoo-Related Side Effects

The risk of infection is influenced by various factors, including the skin’s condition at the tattoo site, the proper sterilization of equipment, the use of contaminated tattoo ink, inadequate disinfection of the tattooed skin area, and inappropriate aftercare [3,45,46]. During the healing process of the injured tissue after tattooing, patients often experience pruritus and burning, which increase the risk of superinfection due to scratching and the subsequent introduction of microorganisms [3,45,46].

Infections on tattoos can manifest either as pyogenic or nonpyogenic. In contemporary times, due to standard hygiene practices and modern aseptic tattooing techniques, the majority of infections are typically superficial (acute superficial pyogenic infections, including folliculitis, impetigo, and ecthyma), of bacterial origin, and manifest within a few days post-tattooing [3,7,47]. One Danish study revealed that 10% of the unopened tattoo ink stock bottles were contaminated with a range of bacteria, including both pathogenic and nonpathogenic strains [47,48]. Examples of isolated strains include *Pseudomonas* species, *Staphylococcus* species, *Streptococcus salivarius*, *Streptococcus sanguinis*, *Enterococcus faecium*, and *Acinetobacter* species [47,48]. Additionally, 28% of the analyzed stock bottles were found to be inadequately sealed [47,48].

However, more severe systemic infections can also occur, such as cellulitis, furunculosis, necrotizing fasciitis, erysipelas, or bacterial endocarditis [49,50,51,52,53,54,55]. Historical records of gangrene, tetanus, amputations, and syphilis have also been documented [49,50,51,52,53,54,55].

#### 3.2.1. Bacterial Infections

The most commonly encountered clinical infections related to tattoos include impetigo and folliculitis [3,55]. *Staphylococcus aureus*, *Streptococcus pyogenes*, *Clostridium difficile*, and *Pseudomonas aeruginosa* are the primary causative agents for these superficial infections [3,55] (Table 2).

Clinical manifestations of bacterial infections encompass local pain, erythema, and swelling, as well as fever and purulence [3,56]. It is crucial to differentiate cellulitis or erysipelas from temporary tattoo-induced edema, which is a transient reaction inherent to the tattooing process, particularly when applied to the lower extremities [3,56]. This reaction is inevitable and can occur in any individual [3,56].

Most bacterial infections are easily treatable, and their treatment generally aligns with standard bacterial infection management (Table 2). They can be verified through suitable cultures and subsequently treated accordingly. However, certain pathogens may pose greater challenges. For instance, an epidemic of cutaneous infections caused by methicillin-resistant *Staphylococcus aureus* was reported in the USA following tattooing [3,12,57].

**Table 2:** Bacterial and mycobacterial tattoo-related side effects and clinical measures.

Side Effects	Bacterial	Mycobacterial			
Clinical measures	*Staphylococcus aureus/Streptococcus pyogenes/Clostridium difficile/Pseudomonas aeruginosa* [3,55]	*Mycobacterium tuberculosis/Mycobacterium bovis* [3,7,17,58,59]	*Mycobacterium chelonae/Mycobacterium abscessus/Mycobacterium fortuitum* [6,15,16]	*Mycobacterium mageritense* [3,60,61]	*Mycobacterium leprae* [1,3,62]
	Standard bacterial infection management	Multidrug therapy administered in two phases [63]: Isoniazid Rifampicin Pyrazinamide Ethambutol or Streptomycin	Macrolide antibiotics [64] (Clarithromycin) 4 months in mild cases 6–12 months in severe cases	Antibiotic therapy [64]: Amikacin Imipenem Cefoxitin Fluroquinolones Sulfonamides	Paucibacillary disease [65]: Dapsone + Rifampicin + Clofazimine for 6 months
					Multibacillary disease [65]: Dapsone + Rifampicin + Clofazimine for 12 months
					Rifampicin resistance [65]: 24 months treatment broken down as 6 months of Clofazimine + Ofloxacin and Minocycline followed by 18 months of Clofazimine + Ofloxacin or Minocycline
					Dapsone resistance [65]: Clofazimine + Rifampicin for 6 months

In recent decades, one case of secondary syphilis occurring within a tattoo has been reported [3,14]. In the 19th century, syphilis was more frequently described in the context of tattoos [3,14]. During that period, tattoo artists often moistened needles with saliva or used nonsterile or previously used needles, potentially leading to the contamination of patients with *Treponema pallidum* [3,14].

#### 3.2.2. Mycobacterial Infections

Tattoo-inoculated mycobacterial infections encompass tuberculosis, leprosy, and atypical mycobacteria such as *Mycobacterium chelonae* and *Mycobacterium abscessus* [6,15,16].

Tattooing can lead to the development of primary cutaneous tuberculosis [3,7,16,58]. This occurs when individuals lacking previous immunity are inoculated with *Mycobacterium tuberculosis* or *Mycobacterium bovis* [3,7,17,58,59]. Within 2–4 weeks, an erythematous papule or nodule emerges, eventually progressing to a superficial ulcer known as a tuberculous chancre [3,7,17,58,59]. Often, painless regional lymphadenopathy ensues within 3 to 8 weeks [3,7,17,58,59]. In cases where the patient’s immune system is compromised, there is a risk of progression to lupus vulgaris and tuberculosis cutis verrucose, or even hematogenous spread [3,17,58,59]. Differential diagnoses include foreign-body granuloma, sarcoidosis, inoculation leprosy, tertiary syphilis, and infections with atypical mycobacteria [7]. The histological examination typically reveals epithelioid histiocytes, Langhans giant cells, and tuberculoid granulomas, with or without central caseous necrosis [3,17,58,59]. A positive tuberculin test holds significant diagnostic value for primary tuberculosis [7,17,58,59].

The treatment approach for cutaneous tuberculosis is consistent with that of systemic tuberculosis and involves multidrug therapy [63]. Commonly used drugs include isoniazid, rifampicin, pyrazinamide, and ethambutol or streptomycin, administered in two phases: the intensive one (which aims to rapidly reduce the burden of *Mycobacterium tuberculosis* and typically spans about 8 weeks) and the continuation phase, designed to eradicate any remaining bacteria and extends for a duration of 9 to 12 months [63] (Table 2). Strict adherence to the treatment regimen is crucial for a successful cure [63].

Various factors influence the outcomes of treatment, including the patient’s immunity, overall health, disease stage, type of cutaneous lesions, treatment adherence, duration of therapy, and potential side effects [63].

Atypical mycobacterial infections, particularly with *Mycobacterium chelonae*, appear to be an emerging complication [3,66,67,68,69]. This occurrence is particularly associated with the preparation of grey ink, which is obtained by diluting black ink with water [3,67]. If the water used in this process is contaminated with *Mycobacterium chelonae*, a bacterium commonly found in nonsterile water, it can lead to infections [3,67]. Less commonly, skin infections can be caused by other mycobacterial species, such as *Mycobacterium haemophilum, Mycobacterium abscessus, Mycobacterium immunogenum, Mycobacterium massiliense, Mycobacterium mageritense*, and *Mycobacterium fortuitum* [3,60,61] (Table 2). Interestingly, mycobacterial infections tend to manifest more frequently in the grey or black areas of a tattoo [3,67]. Clinically, lesions present as chronic papules, pustules, lichenoid plaques, and plaques with scales, typically developing within 1 to 3 weeks after the procedure [3,67]. Ulcerated nodules primarily confined to the tattooed area have also been reported [3,67].

For skin and soft-tissue infections caused by nontuberculous mycobacteria, a prolonged treatment regimen involving combination therapy with at least two susceptible antimicrobials is recommended to minimize the risk of antibiotic resistance [64]. Typically, the recommended duration of therapy for mild cases is around 4 months, while severe cases may require treatment for 6–12 months [64]. Macrolide antibiotics, with clarithromycin commonly included, are considered standard treatment for nontuberculous mycobacteria infections, including those associated with tattoos and involving *Mycobacterium chelonae*, *Mycobacterium abscessus*, and *Mycobacterium fortuitum* [64]. However, it is important to note that *Mycobacterium mageritense* is known to be resistant to macrolides due to the presence of the erythromycin ribosomal methylase gene, which imparts resistance to macrolide antibiotics [64]. *Mycobacterium mageritense* generally exhibits susceptibility or intermediate susceptibility to amikacin, imipenem, cefoxitin, fluoroquinolones, and sulfonamides but is resistant to clarithromycin [64]. It is essential to guide antibiotic therapy based on susceptibility testing [64] (Table 2).

Instances of tattoo inoculation with *Mycobacterium leprae* are predominantly reported in regions where leprosy is endemic, and unhygienic tattooing practices are prevalent [1,3,62]. The onset of leprosy after tattooing can vary significantly, occurring between 10 to 20 years post-tattooing [1,3,62]. Outbreaks have been linked to the use of shared needles during unhygienic tattooing by roadside artists [3,7,62]. Manifestation of leprosy skin lesions may occur 10 to 20 years after the initial inoculation, and the clinical presentation is primarily influenced by the immunologic status of the host [3,7,62]. In cases where a mycobacterial infection is suspected, conducting a biopsy, tissue culture, and polymerase chain reaction for Mycobacterium species is recommended [3,7,62]. Histologically, these reactions are characterized by the formation of suppurative granulomas with the presence of polymorphonuclear leukocytes [3,7,62].

Treatment recommendations for leprosy in adults consist of long-term multidrug therapy: dapsone, rifampicin, and clofazimine for 6 months in paucibacillary disease and for 12 months in case of multibacillary disease [65]. In case of rifampicin resistance, clofazimine plus at least two of minocycline, clarithromycin, and quinolone for 6 months is recommended, followed by an additional 18 months of clofazimine plus one of the aforementioned drugs [65] (Table 2).

#### 3.2.3. Viral Infections

**Figure 6:**
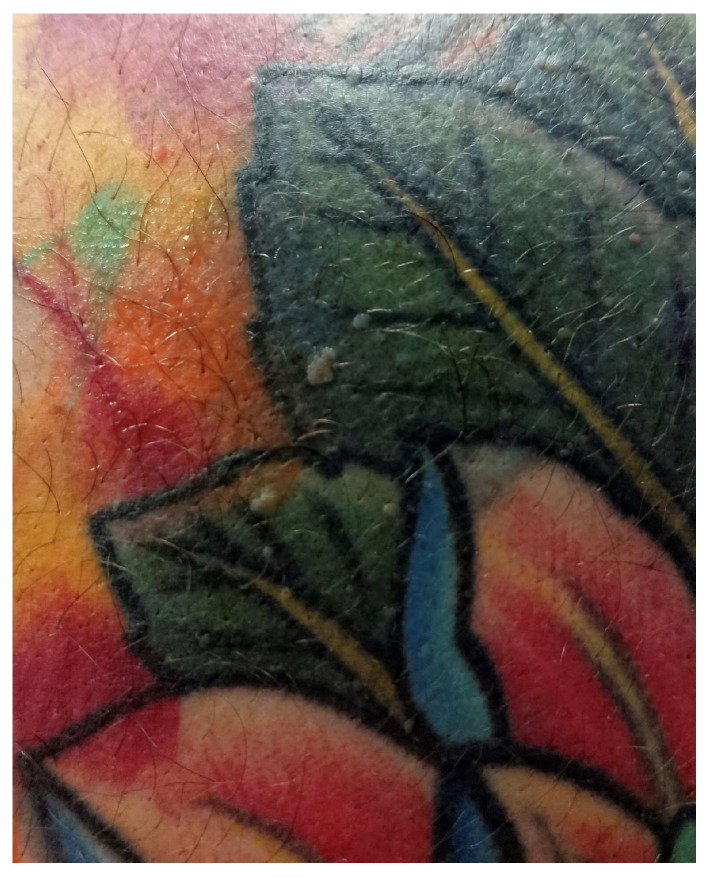
Multiple viral warts localized on the trunk.

The transmission of infections such as verrucae, molluscum contagiosum virus, human papillomavirus (HPV), herpes simplex virus (HSV), human immunodeficiency virus (HIV), and hepatitis B (HBV) and C viruses (HCV) has been documented [3,7] (Figure 6 and Table 3).

**Figure 7:**
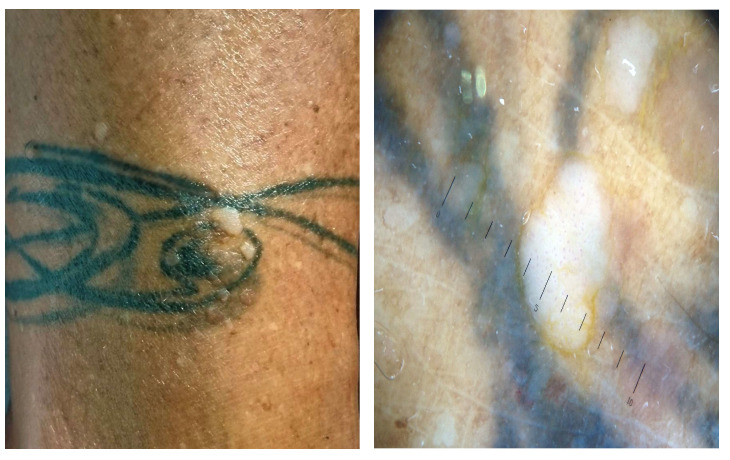
Clinical and dermoscopic features of viral warts localized on the right leg.

**Figure 8:**
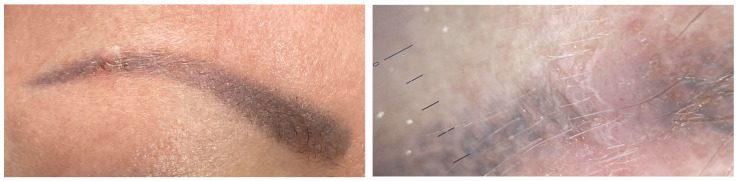
Clinical and dermoscopic features of a viral wart in a microtattooed eyebrow.

Viral warts and molluscum contagiosum lesions exhibit varying numbers and sizes, sometimes confined to a specific tattoo-ink color [3,52,70,71,72] (Figure 7). Onset may occur between 1 month and 10 years after tattooing [3,52,70,71,72] (Figure 8). The inoculation may be associated with contaminated instruments, alterations in local immunity related to the ink, or intense UV-light exposure [3,52,70,71,72]. However, the most plausible hypothesis remains the pre-existence of microscopic skin lesions disseminated through the tattoo drawing by a Koebner phenomenon [3,52,70,71,72]. When multiple viral lesions spontaneously appear within a tattoo, it may prompt testing for underlying immunodeficiencies [3,92].

First-line treatment approaches for viral warts are salicylic acid and cryotherapy [75,76]. Refractory warts could benefit from topical immunotherapy with contact allergens, intralesional bleomycin, and fluorouracil [76] (Table 3). A variety of other additional treatments include cantharidin, imiquimod, trichloroacetic acid, pulsed dye laser, intralesional immunotherapy, and surgery [75,76,86,87,88,89,90,91] (Table 3).

First-line therapies for molluscum contagiosum lesions include cryotherapy, curettage, cantharidin, and podophyllotoxin [77,78,79] (Table 3). Other treatment considerations involve imiquimod, salicylic acid, and topical retinoids [77,78,79,81,82,83,84,85] (Table 3).

Isolated cases of HPV and HSV within tattoos have been reported. HSV has been documented in people with cosmetically tattooed lips. These infections can either be transmitted during tattooing or reactivated from a previously dormant virus [3,7]. The incubation period typically spans weeks to months [3,7]. The triggering factor may be represented by a recent sunburn, suggesting that UV radiation could induce immunosuppression and activate HPV [3,7].

Severe viral infections, including HIV, HBV, and HCV have been reported in association with tattooing, the majority of these reports involving tattoos performed in nonprofessional settings [3,7]. With current hygiene regulations and tattoos administered by professional artists, the transmission of these viral infections is considered unlikely [3,6]. Additionally, many individuals with HIV, HBV, or HCV have other potential modes of transmission, such as injection drug use [3,7].

Antivirals represent the standard therapeutic approach, and the involvement of multidisciplinary medical personnel is advisable (Table 3).

#### 3.2.4. Fungal Infections

Fungal infections following tattooing are infrequent. However, there have been rare cases of infections involving dermatophytes, *Aspergillus fumigatus*, sporotrichosis, zygomycosis, *Acremonium fungi*, or Candida [3,7,14,73,74]. The possibility of fungal infections should be taken into consideration when cutaneous complications worsen with the use of topical corticosteroids [3,7,14,73,74].

Antifungals, either systemic (amphotericin B, itraconazole, fluconazole, voriconazole, terbinafine, and griseofulvin) or topically applied (clotrimazole, econazole, miconazole, ketoconazole, nystatin, and terbinafine) represent the standard therapeutic approach (Table 3).

#### 3.2.5. Parasitic Infections

Cases of cutaneous leishmaniasis emerging in tattoos are seldom documented, and all reported ones have been observed in individuals already diagnosed with visceral leishmaniasis or HIV, conditions associated with immunosuppression [3]. The reuse of needles may represent a potential mode of transmission [3].

Diagnosis of cutaneous leishmaniasis relies on a meticulous assessment of the patient’s medical history and a detailed examination of the lesion’s clinical characteristics [80]. In nonendemic areas, obtaining a comprehensive travel history is imperative, given the prolonged incubation period [80]. Confirmation of the diagnosis entails the identification of the parasite through procedures such as biopsy or split skin smear [80]. For a precise determination of the Leishmania species, especially in cases involving a risk of mucocutaneous leishmaniasis, culture and polymerase chain reaction (PCR) techniques are employed [80].

Therapy options include cryotherapy, photodynamic therapy, imiquimod, and intralesional or systemic antimonials (sodium stibogluconate, meglumine antimoniate) [80] (Table 3). Other systemic employed therapies involve amphotericin B, miltefosine, pentamidine, antifungal drugs (itraconazole, fluconazole, ketoconazole), paromomycin, zinc sulfate, and allopurinol [80].

### 3.3. Neoplasms

**Figure 9:**
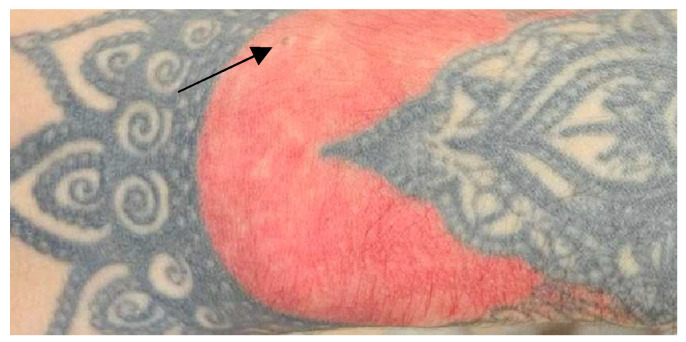
Clinical picture of a hardly identifiable nevus localized on the lateral side of the wrist.

Carcinogenesis is a complex process influenced by various factors contributing to the occurrence of neoplasms in tattooed areas [3,93]. These factors include the intradermal injection of potentially carcinogenic substances (benzopyrene), trauma induced by the tattooing procedure, chronic inflammatory response to foreign material in the skin, UV radiation, and, notably, genetic predisposition [1,3,93]. Additionally, a delayed diagnosis may be present, as tattoos can hide the appearance of new skin lesions and the development or alteration of neoplasms, complicating the clinical evaluation of the skin and potentially causing a delayed diagnosis [3,93]. Moreover, tattooing over a nevus might induce trauma, induce dysplasia, or potentially mask associated dysplastic signs [3,93] (Figure 9).

**Figure 10:**
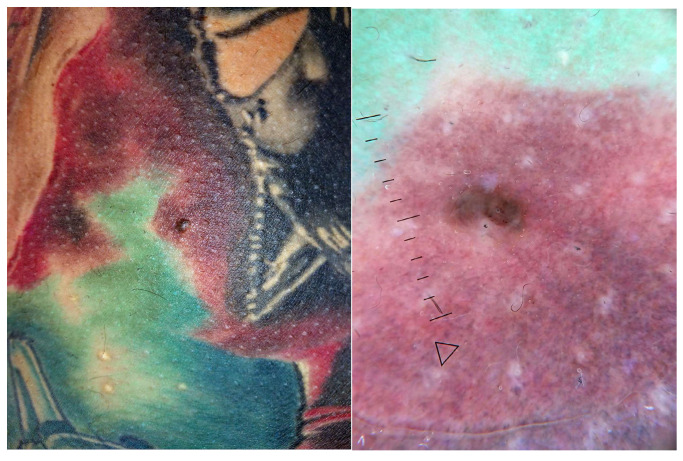
Clinical and dermoscopic features of seborrheic keratosis localized on the left forearm.

**Figure 11:**
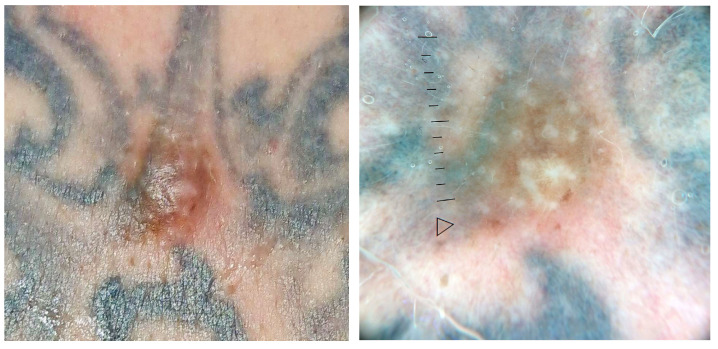
Clinical and dermoscopic features of a dermatofibroma localized on the left leg.

Benign lesions such as seborrheic keratosis (Figure 10), histiocytofibroma, dermatofibroma (Figure 11), epidermal cysts, and milia are commonly observed after tattooing but seldom documented [3,7,46,94,95].

Several cases of melanoma, basal cell carcinoma (BCC), squamous-cell carcinoma (SCC), and keratoacanthoma (KA) in tattoos have been documented [3,95]. Additionally, tattoo ink is believed to contain potential carcinogenic substances, such as aromatic amines and polycyclic aromatic hydrocarbons [55]. Apart from melanoma, BCC, SCC, and KA, isolated cases of rare cutaneous malignancies, including dermatofibrosarcoma protuberans, cutaneous leiomyosarcoma, and cutaneous lymphoma, have been reported [3]. Kluger and Koljonen demonstrated that melanomas and BCCs are more commonly associated with dark-colored tattoos, while SCCs, KAs, and pseudo-epitheliomatous hyperplasia mainly occur on red tattoos [3,93,95].

Lastly, the laser removal of tattoos can pose challenges, as tattoo pigments phagocytosed by macrophages are transferred to regional lymph nodes, potentially creating confusion with metastatic changes [1]. In cases where concurrent melanoma is evident, a histologic pigment analysis is recommended [1].

To confirm the diagnosis, a skin biopsy is essential, as neoplastic conditions may not be readily identified through clinical examination alone. The surgical excision of tumors is undertaken based on the location and dimensions of the lesion, potentially involving lymph node removal in the presence of metastases [1].

### 3.4. Miscellaneous Complications

#### 3.4.1. Neuro-Sensory Complications

Occasionally, unexplained pain or itching in a tattoo has been noticed, and in such cases, clinical and histological abnormalities are typically identified [3]. Morte et al. reported complex regional pain syndrome in an individual with a wrist tattoo [3,96]. It was hypothesized that substances in the ink may have influenced the C-fibers of the sensory nerve due to the tattoo’s location in the proximity of the superficial presence of the cutaneous branch of the median nerve at the wrist [3,96].

#### 3.4.2. Skin Side Effects following Magnetic Resonance Imaging

Notably, tattoos may pose challenges in medical diagnostic studies, leading to evolving issues with procedures such as sentinel lymph nodes, magnetic resonance imaging (MRI), and positron emission tomography (PET) scans [7]. Numerous scientific studies highlighted that patients with tattoos or permanent makeup encountered cutaneous reactions after MRI, including skin irritation, swelling, and burning [3,97,98]. Additionally, tattoos, particularly those with metallic pigments in permanent makeup, may disrupt MRI quality and lead to image artifacts [3,97,98]. This issue tends to arise when pigments containing magneto-ferrous compounds are utilized [3,97,98]. While the precise causative mechanism is unclear and given the fact that symptoms are transient and relatively minor, individuals should not be discouraged from undergoing MRI [3,97].

#### 3.4.3. Photo-Induced Cutaneous Complications

Photosensitive reactions are commonly documented, as indicated by previous reports [2]. Reactions to ultraviolet (UV) light are primarily observed in yellow tattoos, where the swelling response to cadmium sulfide may exhibit phototoxicity [3,7,17]. Among 24 patients with yellow tattoos, 18 experienced edemas in the tattooed area after sun exposure, with four exhibiting a similar reaction in regions colored with red pigment due to the fact that small quantities of cadmium are introduced to enhance the vibrancy of the red tattoo pigment [17].

Photo-induced reactions to cadmium sulfide result in erythematous and edematous lesions in experimentally tattooed areas exposed to light with wavelengths of 380, 400, and 450 nm [3,17]. However, van der Bent et al.’s investigation revealed a comparatively low incidence of patients experiencing (nonallergic) photosensitive reactions [2]. This could be attributed to the generally acute and mild nature of the symptoms, potentially leading patients with only mild complaints to not refer to a dermatologist [2].

The exact pathology of these reactions is not well understood, but they are considered phototoxic and should be treated accordingly [3,17]. Due to the significant shift in tattoo inks and pigments from inorganic to organic (mainly composed of azo dyes), these phototoxic reactions are less frequent nowadays [3,17].

Hutton Carlsen et al. conducted a study directly engaging sunbathing individuals with tattoos on the beach [3,99]. Interestingly, 52% of those approached identified sunlight as the triggering factor for tattoo irritation [3,99]. Sunlight-induced reactions were predominantly reported with red tattoos and exhibited a rapid on-and-off pattern, sometimes occurring within seconds [3,99]. The presumed causative mechanism for these phototoxic reactions involves the photochemical reaction of pigment-inducing reactive oxygen species (ROS) [3,99]. ROS may interact with DNA, proteins, or lipids, compromising their normal functioning and leading to symptoms such as pain, itching, or even cell death [3,99]. Protective measures could include covering tattoos from UV light or using sunscreen [3,99].

Granulomatous reactions occurring incidentally in tattoos becoming visible under blacklight or UV light have been reported as well [3,99,100]. These tattoos, which are hidden until exposed to ultraviolet A (UVA) light (“black light”), may contain polymethylmethacrylate, which fluoresces when exposed to UV light [3,98,99,100]. In these inks, polymethylmethacrylate microspheres are laden with a fluorescent dye. The safety of such tattoo inks remains unclear. Symptoms may resolve after using sunscreen and covering the affected skin area [3,99,100].

### 3.5. Cosmetic Issues

The most common cosmetic adverse reactions often involve dissatisfaction with the tattoo, stemming from issues like misapplication, pigment migration, or pigment fanning [101,102]. Pigment migration has been noted following local anesthetic injections before laser tattoo removal [101,102]. Multiple injections may create tunnels in the skin, allowing ink to spread into the surrounding areas [101,102]. The best cosmetic outcomes are typically achieved with pigment lasers; but caution is demanded, as these lasers can induce irreversible paradoxical darkening of the skin [101,102]. When exposed to laser light, ink containing ferric oxide undergoes irreversible darkening, leading to potentially disfiguring consequences, particularly in permanent makeup [101,102].

A tattoo blowout refers to an adverse reaction where the tattoo pigment disperses beyond the boundaries of the original tattoo due to the ink being injected too deeply into the subcutaneous fat [3,14,103]. This issue may manifest shortly after the completion of the tattoo. It is important to distinguish it from natural aging, which leads to blurry outer lines of a tattoo over time, as a tattoo blowout occurs much more rapidly [3,14,103].

**Figure 12:**
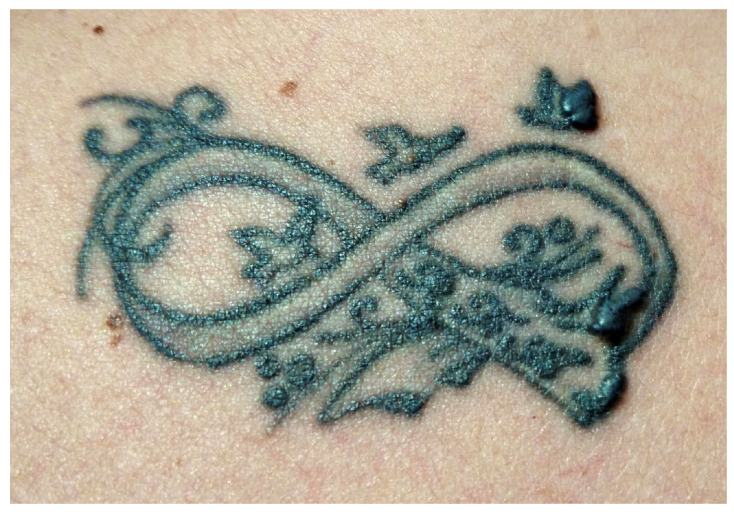
Development of a keloid scar after tattooing on the upper arm.

The presence of tattoo inks may influence the local environment, potentially resulting in an altered immune response and a modified wound-healing process in areas of tattooed skin [10]. Tattooing involves a significant skin trauma that might lead to hypertrophic scars or keloids [2,3,104] (Figure 12). Nevertheless, caution is recommended, particularly for individuals with a history of scars, especially when tattooing predisposed areas such as the upper arms, shoulders, neck, knees, ankles, and sternal area [2].

## 4. Diagnostic Implications

As previously mentioned, assessing pigmented lesions on tattooed skin represents a diagnostic challenge for dermatologists, since a cutaneous lesion might be partially or entirely concealed by tattoo pigment [105,106,107]. Several diagnostic tools may become extremely useful in these particular situations.

A retrospective study was undertaken to identify pigmented lesions located on or near tattooed skin, which were subsequently assessed using reflectance confocal microscopy (RCM) [107]. Reilly et al. concluded that the presence of tattoo pigment did not impede the assessment and diagnosis of pigmented lesions with RCM [107]. As a result, RCM could represent a valuable diagnostic tool for pigmented lesions located on or in proximity to tattooed skin [107].

Melanocytic nevi can be accurately categorized using high-frequency ultrasound (HF-US), demonstrating a robust correlation with both dermoscopic and clinical classifications [108]. HF-US has the potential to unveil the internal morphological characteristics of nevi, thereby aiding in more precise classification and management [108].

Aesthetic procedures may also be influenced by tattoos, especially in the case of microblading eyebrows. In facial regions, the proximity of various muscles increases the risk of complications in botulinum toxin injection procedures, particularly when administered by individuals lacking professional expertise. Therefore, customization of minimally invasive aesthetic procedures through ultrasound imaging is advisable [109].

## 5. Tattooing as a Matter of Global Health

Tattooing, as a form of self expression, has been a ritualized practice across diverse cultures for centuries, and its symbolic significance has evolved both individually and culturally [110]. Encouraging clinicians to engage with individuals potentially considering getting a tattoo may offer them valuable insights into the associated risks and how to make an informed decision [110].

Education extends beyond merely sharing information about various treatments [110]. It includes building relationships and fostering trust [110]. Demonstrating a commitment to educating people and addressing cutaneous adverse reactions linked to tattooing directly empowers people to make informed decisions regarding their future options. People should refer to a dermatological consultation before getting a tattoo, in which an extensive evaluation of associated skin diseases and comorbidities should be performed along with a dermoscopic evaluation. As it is not uncommon for cutaneous tattoo reactions to serve as the initial presentation of an underlying disease, individuals with autoimmune skin conditions should be aware of these potential adverse reactions before undergoing any tattoo procedure [110].

The prevention of the majority of tattooing complications relies on meticulous adherence to already-established guidelines [110]. Strict aseptic precautions are imperative to forestall viral, bacterial, and fungal infections. It is essential that instruments maintain sterility, with a preference for disposables to mitigate the risk of transmissible infections such as HBV, HCV, HIV, and leprosy [110]. Additionally, diverse countries enforce regulations governing blood donation post-tattooing, with varying periods typically ranging from 4 months to 1 year [110].

Thoroughly cleaning the skin to be tattooed is mandatory to prevent the introduction of resident skin organisms into the dermis [110]. Inks should be sterile and of high quality, devoid of extraneous contaminants, to minimize the incidence of allergic and granulomatous reactions [110]. Tattooing should be performed by trained personnel to ensure proper pigment placement at the appropriate depth [110].

Nevertheless, specific tattoo inks are available to facilitate easy removal [10]. They contain bioresorbable dyes encapsulated in polymethylmethacrylate beads, with pigments designed to permit targeting of the tattoo by specific laser wavelengths [10].

Effective communication between customers and tattoo artists is also important and may be achieved by means of trust, credibility, and dedication to delivering the highest quality care [110]. Identification of distinct customer segments allows for tailoring educational initiatives in order to meet the unique needs of each person [110]. Both customers and tattoo artists should be aware of the possible side effects of tattooing, as it represents a matter of global health.

## 6. Conclusions

The prevalence of decorative tattooing has markedly increased as a popular form of body art, especially among young adults. Presently, there is a notable surge in tattooing, with a focus on cosmetic and decorative aspects. However, there is a deficiency in strict requirements, regulations, and legislative measures to guarantee the safety of tattoo procedures. As a result, there has been a growing number of reported adverse reactions following tattooing.

While many of these reactions are generally not life threatening, a consideration of potentially serious skin conditions is essential. The adverse reactions can be categorized into five main groups: inflammatory reactions, infections, neoplasms, miscellaneous, and others. While infectious diseases are directly linked to the tattooing process and can be mitigated through the education and training of tattoo professionals, other complications are often less foreseeable.

By actively engaging in the education of individuals and addressing the cutaneous adverse reactions associated with tattooing, there is a direct empowerment for people to make well-informed decisions regarding their future choices. It is recommended that individuals seek a dermatological consultation before getting a tattoo, encompassing a comprehensive assessment of related skin diseases and comorbidities, along with a dermoscopic evaluation. Given the occasional occurrence of cutaneous tattoo reactions serving as the primary manifestation of an underlying disease, individuals with autoimmune skin conditions should be mindful of these potential adverse reactions prior to undergoing any tattoo procedure, particularly when the dermatosis is active. Moreover, enhanced regulatory oversight in ink manufacturing is crucial to prevent the introduction of toxic, carcinogenic, or immunogenic substances. Nevertheless, the true incidence of adverse reactions related to tattoos is still challenging to estimate due to the lack of comprehensive data.

## Figures and Tables

**Table 1:** Inflammatory tattoo-related side effects and clinical measures.

Side Effects	Allergic Reactions [10]		Autoimmune Dermatoses and Auto-Inflammatory Afflictions [3,7,11,12,13]
Clinical measures	Conservative [1]	Invasive [1]	Standard treatment according to each affliction separately
	Oral antihistamines	Cryotherapy	
	Sunlight exposure avoidance	Electrosurgery	
	Topical steroids: *Clobetasol propionate* [1,5] Oral steroids Intralesional steroids [5]	Punch biopsies	
		Surgical excision Dermabrasion Chemical destruction: acids, ablation, non-Q-switched CO_2_ laser	

**Table 3:** Viral, fungal, and parasitic tattoo-related side-effects and clinical measures.

Side Effects	Viral			Fungal	Parasitic
Clinical measures	Viral warts [3,52,70,71,72]	*Molluscum contagiosum* [3,52,70,71,72]	HPV, HSV, HIV, HBV and HCV [3,7]	*Dermatophytes/Aspergillus fumigatus/Sporotrichosis/Zygomycosis/Acremonium fungi/Candida* [3,7,14,73,74]	Leishmania species [3]
	Firstline [75,76]: Salicylic Acid Cryotherapy	First-line [77,78,79]: Cryotherapy Curetage Cantharidin Podophyllotoxin	Multidisciplinary medical personnel (infectious disease specialist) Antivirals as standard therapeutic approach	Topical antifungals: Clotrimazole Econazole Miconazole Ketoconazole Nystatin Terbinafine	Cryotherapy Photodynamic therapy Imiquimod [80]
	Refractory warts [76]: Topical immunotherapy (contact allergens, intralesional Bleomycin, Fluorouracil)	Other [77,78,79,81,82,83,84,85]: Imiquimod Salicylic Acid Topical retinoids		Systemic antifungals: Amphotericin B Itraconazole Fluconazole Voriconazole Terbinafine Griseofulvin	Intralesional or systemic antimonials [80]: Sodium stibogluconate Meglumine antimoniate
	Other [75,76,86,87,88,89,90,91]: Cantharidin Imiquimod Trichloroacetic acid Pulsed dye laser Intralesional immunotheraphy Surgery				Other systemic therapies [80]: AmphotericinB Miltefosine Pentamidin Itraconazole Fluconazole Ketoconazole Paromomycin Zinc sulfate Allopurinol

## Data Availability

This review summarizes data reported in the literature, and it does not report primary data.
